# Effectiveness, retention, and safety of modified ketogenic diet in adults with epilepsy at a tertiary-care centre in the UK

**DOI:** 10.1007/s00415-019-09658-6

**Published:** 2020-01-10

**Authors:** S. F. Green, P. Nguyen, K. Kaalund-Hansen, S. Rajakulendran, Elaine Murphy

**Affiliations:** 1 Epilepsy Department, The National Hospital for Neurology and Neurosurgery, Queen Square, London, WC1N 3BG UK; 2 Charles Dent Metabolic Unit, The National Hospital for Neurology and Neurosurgery, Queen Square, London, WC1N 3BG UK; 3 University College London, London, UK

**Keywords:** Epilepsy, Seizures, Ketogenic diet, Ketones, Adult

## Abstract

With the rising demand for ketogenic diet therapy in adult epilepsy, there is a need for research describing the real-life effectiveness, retention, and safety of relevant services. In this 1-year prospective cohort study we present outcomes of the first 100 referrals for modified ketogenic diet (MKD) at the UK’s largest tertiary-care epilepsy centre, where patients received dietetic review up to twice per week. Of the first 100 referrals, 42 (31 females, 11 males; mean age 36.8 [SD ± 11.4 years]) commenced MKD, having used a mean of 4 (SD ± 3) previous antiepileptic drugs. Retention rates were: 60% at 3 months, 43% at 6 months, and 29% at 12 months. 60% of patients reported an improvement in seizure frequency, 38% reported a > 50% reduction, and 13% reported a period of seizure freedom; 30% reported a worsening in seizure frequency at some point during MKD therapy. The most common reasons for discontinuing MKD were side effects and diet restrictiveness. The most common side effects were weight loss, gastrointestinal symptoms and low mood. The likelihood of discontinuing MKD was significantly decreased by experiencing an improvement in seizure frequency (*p* ≤ 0.001). This study demonstrates that MKD can be effective in adults, although, even with regular dietetic support, retention rates remain low, and periods of worsening seizure frequency are common.

## Introduction

Approximately one third of adult epilepsy patients do not achieve seizure freedom with antiepileptic drugs (AEDs) [1, 2]. Those who have failed on two AEDs have been found to have a < 5% chance of achieving seizure freedom with a further AED trial [3]. These patients with drug-resistant epilepsy carry a significant disease burden [4, 5]. In addition, many are either not amenable to or decline surgical intervention [6], further underpinning the need for effective, alternative treatment options.

Ketogenic diet therapy, characterised by a high fat and low carbohydrate diet, is widely used for drug-resistant epilepsy in children, for whom there is a strong evidence base for efficacy [7, 8]. Whilst the ‘classic’ ketogenic diet involves a strict 3:1–4:1 ratio of fats to combined carbohydrate and protein, a number of variations have been developed in an attempt to improve tolerability and reduce adverse effects. These include the modified Atkins diet (MAD), low glycaemic index treatment, and the modified ketogenic diet (MKD). Carbohydrate restriction is similar between the MAD and MKD, however, in the MKD, there is a target amount of fat (approximately 80% of calories) to ensure daily calorie intake is controlled. The mechanism behind the putative antiepileptic effects of dietary manipulation remains unclear (for a review, see [9]).

Feasibility studies have endorsed the use of ketogenic diets in adults [10], however, access to appropriate services within the UK remains limited. There is an increased demand for such services [11], and more centres worldwide are prescribing ketogenic diets than before [12]. A recent meta-analysis showed that 53% of patients with intractable epilepsy can achieve a reduction in seizure frequency of > 50% with a ketogenic diet [13]. However, its effectiveness is limited by the availability of suitably trained dietitians [14], poor compliance [15], lack of efficacy in certain circumstances [16], and side effects [16]. Despite the success of ketogenic diets in a controlled environment [7], establishing a ketogenic diet service for adults with favourable ‘real-life’ effectiveness, retention and safety remains a significant challenge [17].

We present outcomes of the first 100 referrals to a dedicated ketogenic diet service for adults with drug-resistant epilepsy at the largest epilepsy centre in the UK. We investigated the real-life feasibility of this service and describe observational data on the effectiveness, retention rate and safety profile of the MKD.

## Methods

### Study design and data collection

This was a prospective cohort study of the first 100 referrals made to the Adult Ketogenic Diet Service at the National Hospital for Neurology and Neurosurgery, Queen Square, London, with patients commencing MKD between January 2016 and January 2018. The follow-up period was 12 months. Data were collected from regular (between twice per week and once per fortnight) telephone consultations with a dietitian, and baseline and follow-up appointments every 3 months with a clinician. Data were collected on the following: number initiating treatment, reasons for not initiating treatment, length of adherence, reasons for discontinuation, side effects, number of previous AEDs, number of current AEDs, seizure types and epilepsy syndrome, coexistent and previous epilepsy treatments (e.g. vagus nerve stimulation or epilepsy surgery), and seizure frequency. Side effects were self-reported by study participants.

Effects on seizure frequency were categorised to determine whether the patient experienced: (a) > 50% reduction in seizure frequency from pre-MKD baseline (often labelled as ‘responders’), (b) seizure freedom, defined as a period of freedom from all types of seizures for three-times the longest pre-diet interval between seizures [18], (c) any improvement in seizure frequency, including a reduction in a specific seizure type, or a reduction in the frequency of seizure clusters, (d) a period of worsening in seizure frequency, (e) other benefits of treatment such as increased mental alertness or faster seizure recovery. Seizure frequency was recorded twice per week during telephone appointments with dietitians for the first 3 months. Then it was recorded once per fortnight with dietitians and in outpatient clinic appointments every 3 months. The categories denoting improvement in seizure frequency were not mutually exclusive. Concomitant changes in AEDs during MKD therapy were also recorded.

### Ethics statement

This manuscript does not contain clinical studies or identifiable patient data. This study was approved as a local service evaluation by the Queen Square (National Hospital for Neurology and Neurosurgery, University College London Hospitals NHS Foundation Trust) Clinical Audit and Quality Improvement Committee.

### Pre-treatment evaluation

Any patient age 18 years or older with diagnosed epilepsy could be referred to the service. Referred patients were evaluated in an outpatient clinic by the MKD team to discuss the diet and assess suitability. Baseline blood and urine tests were taken. Patients were provided with a diet diary. If participants completed a preliminary diet diary and questionnaires (diet-specific unvalidated readiness to change and quality-of-life questionnaires) after the first clinic appointment, they were booked into a group education session during which they were taught the dietary principles of MKD, macronutrient content of foods and how to plan meals to meet their prescribed macronutrient targets. Training on blood ketone and glucose testing was also provided. If patients did not attend their first appointment, they were offered two further opportunities.

### Diet prescriptions

Patients’ estimated energy requirements and macronutrient targets were calculated according to baseline anthropometric data. MKD was prescribed to provide a minimum of 80% of total calories from fat. Between 10 and 25 g of carbohydrates were prescribed per day (approximately 5% of total calories). MKD was introduced over a 1-week period progressively replacing meals.

### Monitoring and follow-up

Patients had telephone consultations with a dietitian twice per week during the initial 12-week period, with monitoring of weight changes, seizure activity, ketone and glucose levels, bowel habit, adherence to dietary prescriptions. General advice was provided to help improve tolerance and maintain compliance. Upon completion of the initial 12 weeks on MKD, patients were seen every 3 months by a clinician and dietitian to review their progress and determine if continuation on MKD was appropriate. Patients who continued on MKD underwent haematology and biochemistry blood tests every 3 months including: full blood count, urea and electrolytes, liver function tests, lipid profile, bone profile, magnesium, carnitine (total, free), and urine calcium excretion.

### Statistical analysis

Using SPSS (SPSS, Inc.), descriptive statistics were produced to measure clinical and demographic baseline characteristics, as well as seizure frequency data. For blood and urine markers, measurements taken at baseline and every 3 months were analysed used a mixed linear model (fixed effects) with a factor of time. Cox regression was used to identify the effects of covariates on retention rates using the Cox proportional hazards model. The following covariates were tested: (a) type of epilepsy, (b) improvement in seizure frequency, (c) gender, (d) side effects. Previous studies have found that type of epilepsy [19], improvement in seizure frequency [20], and side effects [21] may affect retention rates for AEDs and dietary interventions. Previous groups have also shown that ketogenic diet cohorts are predominantly female [22], and we were interested to see whether gender might affect retention rates. To correct for multiple comparisons, the alpha threshold was adjusted by Bonferroni correction. The Chi-square test was used to compare the effects of MKD on seizure frequency between generalised and focal epilepsy syndromes. GraphPad Prism (GraphPad, Inc.) was used to produce a Kaplan–Meier plot to visualise survival curves indicating length of retention to MKD.

## Results

**Fig. 1:**
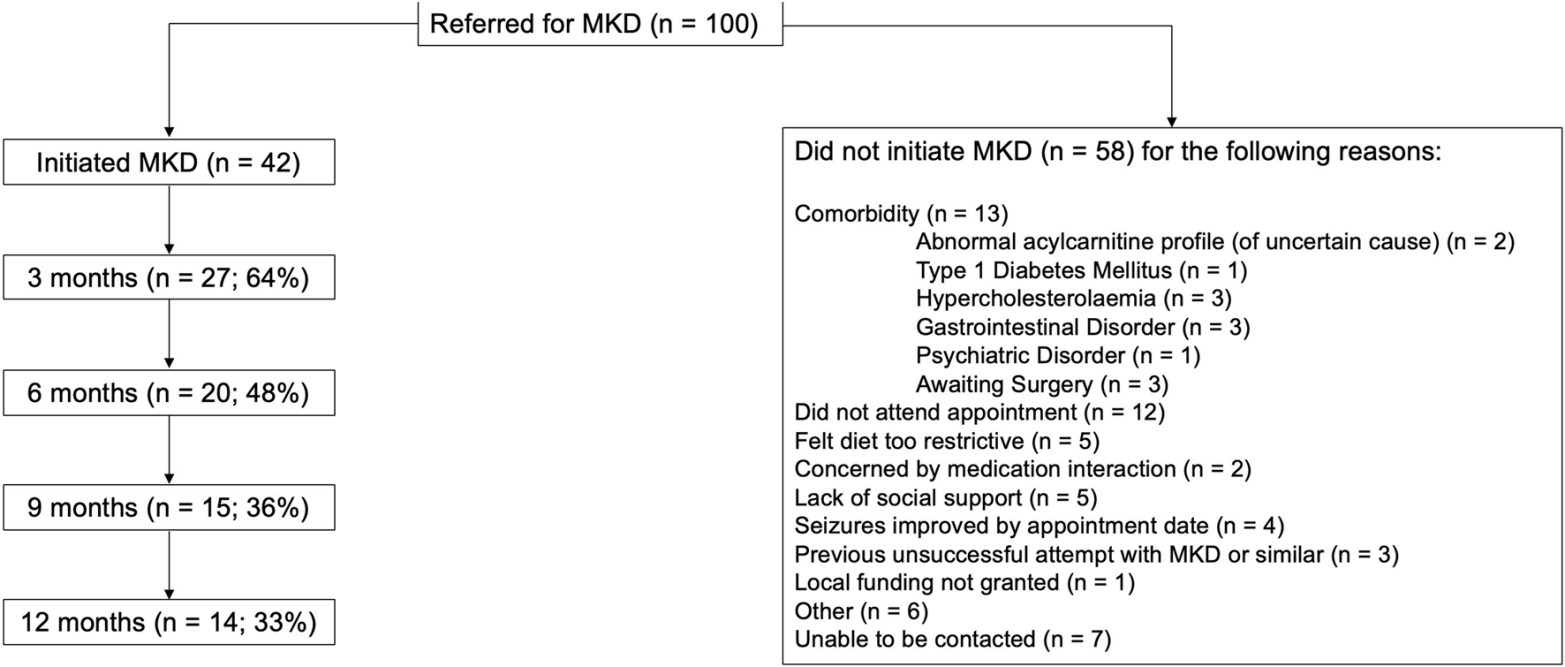
Retention to modified ketogenic diet and reasons for not initiating treatment. Percentages reflect the proportion of patients who started MKD continuing the diet at each 3-month timepoint. “Lack of social support” refers to instances where a patient may be living alone with limited family or friends available to support a trial of MKD, have limited access to food and/or cooking facilities, may have low literacy or numeracy skills, or a learning disability

Of the first 100 referrals to the service, 42 initiated MKD (Fig. 1).

For full baseline clinical and demographic characteristics of the patients who initiated MKD see Table 1. The mean number of years (± SD) between being diagnosed with epilepsy and commencing MKD through the adult ketogenic diet service was 21.0 (9.8). The mean number of current AEDs used was 2.57 (SD 1.45, IQR 1–4, range 0–6), and the mean number of previous AEDs tried was 4.40 (SD 3.35, IQR 1.75–6, range 0–12). Across all patients, the following frequencies of seizure types were observed: tonic–clonic 16/42 (38%); focal to bilateral tonic–clonic 13/42 (31%); focal-onset impaired awareness 27/42 (64%); focal-onset aware 10/42 (24%); absence 8/42 (19%); myoclonic 5/42 (12%); tonic 1/42 (2%), atonic 1/42 (2%); other 3/42 (7%). Full details of previous and current AEDs are described in Table 2.

**Table 1:** Demographic and clinical characteristics of the cohort initiating MKD (*n* = 42)

Characteristics	Mean (SD)
Age	36.8 (11.4)
Age at epilepsy onset	18.9 (12.2)
Number of previous AEDs	4 (3)
Number of current AEDs	3 (2)

Characteristics	*n* (%)
Female/male	31/11 (74/26)
Vagal nerve stimulation	4 (10)
Previous epilepsy surgery	4 (10)
Previous ketogenic diet therapy	8 (19)
Focal epilepsy	26 (62)
Symptomatic	19 (45)
Cryptogenic	7 (17)
Generalised epilepsy	16 (38)
Idiopathic	12 (29)
Symptomatic	3 (7)
Other	1 (2)
Taking AEDs	39 (93)
0	3 (7)
1	8 (19)
2	9 (21)
3 or more	22 (52)

*AED* antiepileptic drug, *MKD* modified ketogenic diet, *SD* standard deviation

**Table 2:** Previous and current antiepileptic drugs used at the point of initiating MKD

Antiepileptic drug	Current (*n*)	Previous (*n*)
Levetiracetam	19	17
Carbamazepine	13	18
Clobazam	13	10
Zonisamide	11	11
Lacosamide	8	10
Lamotrigine	8	19
Clonazepam	6	6
Pregabalin	5	7
Sodium valproate	5	17
Oxcarbazepine	4	6
Other	3	5
Perampanel	3	6
Topiramate	3	19
Eslicarbazepine	2	0
Phenytoin	2	12
Lorazepam	1	0
Phenobarbitone	1	8
Primidone	1	2
Acetazolamide	0	2
Ethosuximide	0	5
Gabapentin	0	2
Retigabine	0	0
Tiagabine	0	1
Vigabatrin	0	2

Two participants were excluded from analysis of seizure frequency. One of these had idiopathic generalised epilepsy and had attained seizure freedom on lamotrigine shortly prior to commencing MKD. Nonetheless, they wanted to commence MKD for its putative benefits on cognition. They were included in retention and side effect data analysis, but not seizure frequency data analysis. A second patient was excluded due to significant concomitant medication changes (started a new AED) within the first few weeks of MKD. This left 40 patients, of whom three (7.5%) made minor concomitant medication changes after the first few months of MKD but were still included in data analysis. These medication changes included: (a) slow weaning of perampanel from 6 to 0 mg per day from weeks 20 to 52; (b) commencement of perampanel between weeks 20 and 32 at 2 mg per day, and increasing lacosamide from 300 to 400 mg total dose per day at week 44; (c) weaning clonazepam by 0.5 mg per day to 0 mg from week 20, up-titrating of oxcarbazepine from 300 to 600 mg three times a day in week 20, and increasing valproate by 300 mg total daily dose in week 24. The analysis of seizure frequency was repeated with the three patients who had minor medication changes excluded, demonstrating improvement in seizures in 57% (21/37), > 50% reduction in seizures in 32% (12/37), seizure freedom in 11% (4/37) and worsening of seizures in 27% (10/37).

Effects of MKD on seizure frequency, other benefits, and side effects are summarised in Table 3. Four patients (out of 25) were seizure-free at 3 months. Only one participant remained seizure-free during the entire study period. Outcomes of the initial ‘responders’ and ‘responder’ rates at each 3-month interval are described in Table 4. The differences in the effects of MKD on seizure frequency between focal and generalised epilepsy syndromes are summarised in Table 5. Amongst those who reported worsening of seizure frequency, one required hospital admission for status epilepticus, and another experienced worsening of seizure frequency whilst attempting to self-wean off the diet.

**Table 3:** Effects of MKD on seizure frequency, other improvements, and side effects

Outcome	Number of individuals (%)
Improvement in seizure frequency	24 (60)
> 50% reduction in seizure frequency	15 (38)
Worsening of seizure frequency	12 (30)
Experienced a period of seizure freedom	5 (13)
Other improvement	22 (52)
Faster recovery after seizures	10 (25)
Increased mental alertness	13 (31)
Improved mood	4 (10)
Side effects	15 (38)
Gastrointestinal^a^	3 (7)
Weight loss	8 (19)
Poor memory	2 (5)
Low mood	3 (7)
Headache	1 (2)
Fatigue	1 (2)
Renal calculi	1 (2)

Data on seizure frequency denote the total number of patients reporting a change at any 3-month interval compared to baseline. Percentages are calculated based on a total of 40 patients included in the data analysis

^a^Defined as any of nausea, vomiting, abdominal bloating, or change in bowel habit

**Table 4:** Responder rates of MKD

Time since initiation of MKD (m)	*n* (%) of responders	Number of subjects remaining on MKD
(a) Longterm outcome of initial responders (those with > 50% reduction in seizure frequency at 3 m)		
3	9 (23)	25
6	6 (15)	18
9	4 (10)	13
12	2 (5)	12
(b) Total responders (those with a > 50% reduction in seizure frequency at each timepoint)		
3	9 (23)	25
6	8 (20)	18
9	6 (15)	13
12	4 (10)	12

This table demonstrates (a) the number of initial responders (those who experienced > 50% reduction in seizure frequency at 3 months) who continued to experience > 50% reduction at each subsequent measured timepoint and (b) the total number of patients who had experienced a > 50% reduction in seizure frequency at each timepoint compared to baseline. Percentages are calculated based on a total of 40 patients included in the data analysis

A ‘responder’ is defined as a patient experiencing a > 50% reduction in seizure frequency

**Table 5:** The effects of MKD in focal vs generalised epilepsy syndromes

	Focal (%) *n* = 26	Generalised (%) *n* = 14	*χ*^2^_df_	*p*
Seizure improvement	65.4	50	0.88_1_	0.350
> 50% improvement	38.5	35.7	0.031	0.866
Seizure freedom	15.4	7.1	2.311	0.129
Seizure worsening	34.6	21.4	0.741	0.391
Other benefit	57.7	42.9	0.781	0.376

The results of the Chi-square test with unadjusted *p* values are described above

**Fig. 2:**
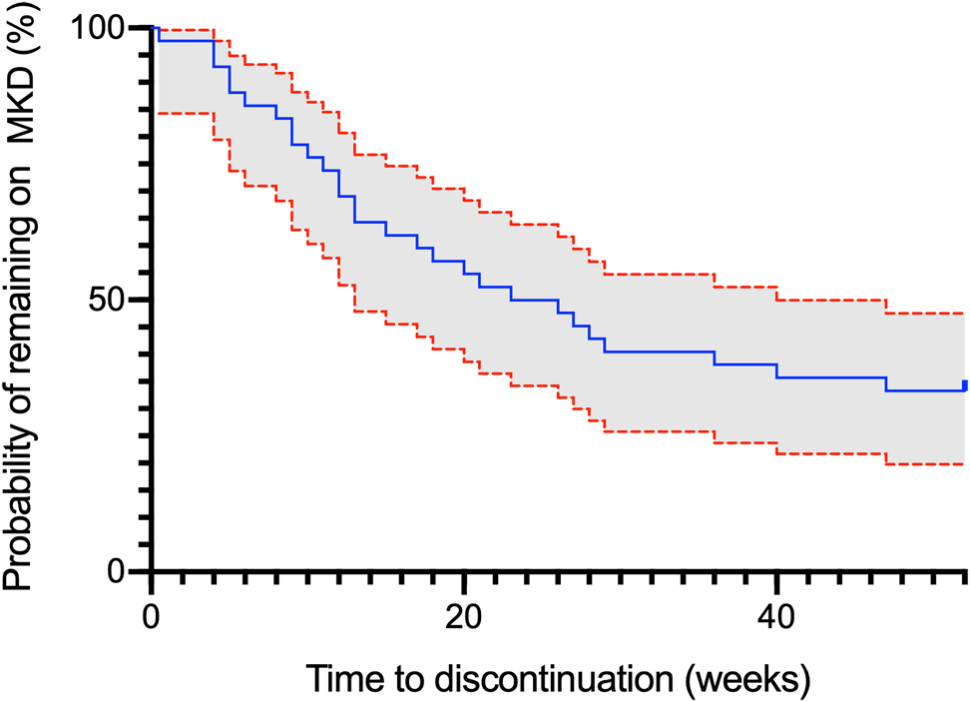
Retention rates of MKD. This Kaplan–Meier plot depicts the time to discontinuation of MKD over the first 12-months. Red lines represent 95% confidence intervals

For retention rates, see Fig. 2. Out of the 28 who did not adhere to MKD for 12 months, reasons reported for discontinuation of diet were as follows: diet too restrictive (8/28, 25%), side effects (10/28, 35.7%), worsening of seizures (4/28, 14.3%), and lack of benefit to seizure frequency (12/28, 42.9%)

Results of the Cox regression showed that there was a significant improvement in fit in the model containing the covariates tested versus the null (*χ*^2^_df_ = 19.07_4_, *p* = 0.001). Experiencing an improvement in seizures significantly decreased the hazard ratio for discontinuing MKD (i.e. increased the likelihood of continuing MKD; hazard ratio [95% CI] 0.201 [0.083–0.485], *p* < 0.001). After adjusting for multiple comparisons (alpha threshold *p* = 0.0125), no other covariate significantly changed the hazard ratio (see Table 6). However, there were trends towards significance for both gender (being female) and experiencing side effects.

**Table 6:** The effects of different covariates on retention rates of MKD

Covariate	Hazard ratio (95% CI)	*p*
Improvement in seizures	0.201 (0.083–0.485)	< 0.001
Female gender	0.421 (0.161–1.103)	0.078
Type of epilepsy (focal or generalised)	0.759 (0.298–1.937)	0.564
Side effects	2.516 (1.117–5.667)	0.026

This table demonstrates the results of the Cox regression analysis, which describes the effects of several covariates on the hazard ratio of discontinuing MKD. *CI* confidence interval. An alpha threshold of *p* = 0.0125 was employed after Bonferroni correction

Results of the mixed linear model showed that there was a significant overall effect of time on levels of total cholesterol, HDL, LDL, cholesterol:HDL ratio, free carnitine and urine calcium:creatinine ratio (see Table 7). There were no other significant changes in blood or urine markers, including triglycerides, haemoglobin, alkaline phosphatase, alanine transaminase, albumin, magnesium, total carnitine, creatinine and calcium. Unfortunately, some patients did not have blood tests completed at every interval. For example, whilst cholesterol levels at 3 months were taken from all 27 patients continuing MKD (100%), only 9 out of 14 patients attended to have cholesterol measured at 12 months. At 3 months, the proportions with elevated total, HDL and LDL cholesterol were 21/27 (78%), 18/27 (67%) and 12/27 (44%), respectively. At 12 months, the proportions with elevated total, HDL and LDL cholesterol were 4/9 (44%), 7/9 (78%) and 2/9 (22%), respectively. Definitions of elevated lipid levels were > 5 mmol/L (total cholesterol), > 1.5 mmol/L (HDL cholesterol) and > 3.5 mmol/L (LDL cholesterol).

**Table 7:** Changes in biochemistry blood and urine markers during the first 12 months on MKD

Marker (unit)	Baseline	3 m	12 m	*F*_*df*1,2_	*p*
Total cholesterol (mmol/L)	5.00 (0.16)	6.27 (0.32)	5.65 (0.31)	6.4944,32.1	0.001
Triglyceride (mmol/L)	1.16 (0.10)	1.39 (0.13)	1.18 (0.12)	0.8754.31.7	0.490
HDL (mmol/L)	1.75 (0.07)	1.97 (0.17)	2.09 (0.11)	2.8054,50.9	0.035
LDL (mmol/L)	2.73 (0.14)	3.70 (0.33)	3.01 (0.30)	4.1734,29.7	0.008
Cholesterol:HDL	3.14 (0.19)	3.78 (0.32)	2.84 (0.20)	4.3354,49.3	0.004
Carnitine (free; µmol/L)	29.6 (1.3)	20.7 (1.7)	22.2 (2.9)	6.1694,22.2	0.002
Urine Ca:Cr	0.54 (0.06)	0.83 (0.11)	0.96 (0.14)	10.6524,23.8	< 0.001

This table demonstrates the results of the mixed linear model (fixed effects) for repeated measures across several time points. Values for each marker are represented as means (standard error of the mean). *F* and *p* statistics correspond to the main effect of time on levels of each biochemistry marker (i.e. describe the overall effect of MKD across all timepoints). Significance threshold *p* < 0.05

*HDL* high-density lipoprotein, *LDL* low-density lipoprotein

## Discussion

This single-centre prospective study investigated the effectiveness, retention and safety profile of a modified ketogenic diet in adults with epilepsy. Overall, MKD showed good effectiveness, improving seizure frequency in 60% of patients, with 38% experiencing > 50% reduction in seizure frequency and 13% experiencing a period of seizure freedom. However, 30% described worsening of seizure frequency during the study period and 67% had discontinued MKD by 12 months. This study adds much-needed observational data on the outcomes of MKD in epilepsy which, like other variations of the ketogenic diet, was employed to optimise the balance between ketosis and palatability. To our knowledge, there is no observational data about the effectiveness of this diet, and outcomes on retention and safety have not yet been reported beyond 3 months [11]. Since adherence to the diet is one of the largest barriers to successfully implementing ketogenic diet therapies [23], more data were needed that describe outcomes of strategies addressing this limitation. This service provided patients with telephone dietetic appointments up to twice per week in addition to regular group education sessions in an attempt to improve adherence.

The proportion achieving a > 50% reduction in seizure frequency in this study is broadly comparable to previous cohort studies of the classic 4:1 [21, 24, 25] or 3:1 ketogenic diet [26]. Compared to studies of MAD, the MKD approach used in this study was less effective at reducing seizures than a review of 206 adults on the MAD, where 49% achieved > 50% reduction in seizures [16]. However, diet effectiveness in this study is comparable to a more recent observational study of MAD in 130 adults [22]. On an ‘as treated’ basis, this includes a similar 3-month responder rate (of 36%) and seizure freedom rate (of 16%), although only one participant (of 40 who commenced MKD) remained seizure-free for 1 year in our cohort, compared to 13 (out of 106 who commenced MAD). Differences between these observational studies likely reflect variations in sample sizes, definition of seizure freedom, epilepsy syndromes [27–29] and dietary differences. Variations in intake of medium-chain triglycerides (MCTs), which may mediate the antiepileptic properties of ketogenic diets might also play a role [30, 31].

Whilst the benefits of MKD on seizure frequency described above may support its use as an intervention in refractory epilepsy, it has previously been observed that as many as 17% of patients with refractory epilepsy achieve seizure freedom by continuing their usual medical therapy [32]. In addition, we note that the proportion experiencing worsening of seizure frequency was relatively high in this study at 30%, particularly when compared to initiation of new AEDs [33]. This proportion experiencing a worsening in seizure frequency during the study period is consistent with previous dietary studies [16, 22, 34]. Reasons for worsening in seizure frequency may include interactions with AEDs [35], variations in compliance with AEDs, or spontaneous fluctuations in underlying seizure frequency, as has been commonly observed at the initiation of both new AEDs and placebo [36].

Relative to recently described large MAD cohorts [15, 22], we had a low retention rate of 33% at 1 year. Retention to ketogenic diets is generally poor [37], despite modifications such as the mixed MAD/classical ketogenic diet [15], owing to various factors including palatability, side effects or lack of benefit [16]. This limits its usefulness in the clinical setting. Lower retention rates in our study most likely reflect the additional challenge of calculating and weighing two variables; both fat and carbohydrate portions, and perhaps the relative palatability of MKD compared to MAD, since effectiveness and side effect profiles are similar. For example, the side effects we report are comparable to both the classical KD and MAD, including GI disturbance and weight loss [16, 34]. These data also reflect the first referrals to a new service. Over time, we feel that with gains in clinical experience, there has been an improvement in retention rates, although this has not been formally measured. We aim to repeat this study once the service is further established.

74% of patients in this cohort were female, but we did not find a significant association between gender and retention to MKD. The high proportion of females in ketogenic diet cohorts is consistent with other studies, such as the Johns Hopkins Adult Epilepsy Diet Center where 71% of diet-naïve participants were female [22]. This group had previously suggested that women may be more likely to want to undergo MKD to achieve the secondary goal of weight loss, which was reported by 19% of our participants. In addition to weight loss, our data support the idea that patients may want to initiate MKD for other reasons than its putative effect on seizure frequency. For example, 31% of participants in this study reported cognitive benefits of MKD, including increased mental alertness and improved mood. It has previously been shown that MKD increases mental alertness, and that this is associated with both a reduction in seizures and diet itself [38].

In addition to decreasing body weight and improving mental alertness and mood, we also found that MKD caused significant derangement in lipid profiles. This included significant increases in total cholesterol, LDL and HDL, but no change in triglycerides. This is in keeping with a previous report of 37 patients [39]. However, this group reported that LDL and total cholesterol returned to baseline by 12 months, as opposed to this study where levels remained elevated. Nonetheless, due to a relatively greater increase in HDL-cholesterol compared with LDL-cholesterol, the cholesterol:HDL ratio is actually lower at 12 months compared with baseline, potentially mitigating any atherogenic impact. It should also be noted that we describe a small sample, as only 9 of 14 patients who reached 12 months on MKD attended to have their lipid profile measured. The longer-term effects of lipid derangement in ketogenic diets on cardiovascular health remain unclear [40].

This study has a few limitations. First, the results did not account for changes or adherence to AEDs, which are likely to have affected seizure frequency, although changes in dose or type of AED were not recommended. Second, patients were included irrespective of their baseline seizure frequency allowing small decreases in seizure frequency to be categorised as large effect sizes, e.g. a > 50% reduction. Third, like most ketogenic diet studies, this was an observational study without a control group, precluding any conclusions being drawn about the efficacy of MKD in epilepsy compared to placebo or usual treatment alone. Fourth, estimating a true baseline seizure frequency was challenging owing to the large range in reported seizures per day or week for some patients. This study is strengthened by having a low risk of recall bias, since seizure frequency was recorded by patients with dietitians up to twice per week.

In summary, this observational study suggests that MKD may be effective as an outpatient treatment option for adults with epilepsy. However, even with intensive dietary support, it remains a challenge for patients to continue the diet long-term, and a significant proportion experience a period of worsening in seizure frequency at some stage. More controlled studies are needed to understand the efficacy of MKD in adult epilepsy compared to other dietary interventions and AEDs.
